# Thalassaemia Intermedia: an Update

**DOI:** 10.4084/MJHID.2009.004

**Published:** 2009-08-29

**Authors:** Ali T. Taher, Khaled M. Musallam, Maria D. Cappellini

**Affiliations:** 1 Department of Internal Medicine, Hematology-Oncology Division, American University of Beirut Medical Centre, Beirut, Lebanon; 2 Universitá di Milano, Policlinico Foundation IRCCS, Milan, Italy

## Abstract

Our understanding of the molecular and pathophysiological mechanisms underlying the disease process in patients with thalassaemia intermedia (TI) has substantially increased over the past decade. TI encompasses a wide clinical spectrum of beta-thalassaemia phenotypes. Some TI patients are asymptomatic until adult life, whereas others are symptomatic from as young as 2 years of age. A number of clinical complications commonly associated with TI are rarely seen in thalassaemia major, including extramedullary hematopoiesis, leg ulcers, gallstones, thrombosis and pulmonary hypertension. There are a number of options currently available for managing patients with TI, including transfusion therapy, iron chelation therapy, modulation of foetal haemoglobin production and haematopoietic stem cell transplantation. However, at present, there are no clear guidelines for an orchestrated optimal treatment plan.

## Introduction:

Thalassaemia was defined as a clinical entity in 1925 by Dr. Thomas B. Cooley at the annual meeting of the American Pediatric Society where he presented five young children with severe anaemia, splenomegaly, and peculiar bone abnormalities^1^. Almost all patients with β-thalassaemia syndromes were later classified as having thalassaemia minor or thalassaemia major (TM). However, a small number of patients did not fit into this categorical distribution. Sturgeon was the first to describe this group in the American literature. He suggested the term thalassaemia intermedia (TI) to describe patients who had clinical manifestations that are too severe to be termed minor and too mild to be termed major^2^. Ever since, our knowledge of the molecular basis and pathophysiology of TI has progressed significantly in the last decade, including an increased understanding of the genetic mutations that lead to the TI phenotypes^3^,^4^. The clinical presentation and sequelae of TI span a wide spectrum of severity. The natural history of the disease allows the emergence of several complications that are relatively unique to TI compared to TM^5^. As such, the optimal course of management for TI patients has been hard to identify and several controversies remain regarding the best treatment plan^6^. This review summarizes molecular and clinical characteristics of TI. Moreover, currently practiced treatment options and insights onto future perspectives are discussed.

## Molecular Understanding

The clinical manifestations of thalassaemia result from defects in one of two types of haemoglobin (Hb) polypeptide chains (alpha or beta). For Hb to function properly, the number of alpha-chains must precisely match the number of beta-chains; thalassaemia is caused by an imbalance in globin chain synthesis. The beta-thalassaemias, including TI, arise from defective gene function leading to the partial suppression of beta-globin protein production. The extent of suppression varies from patient to patient and dictates the clinical severity of disease. Most TI patients are homozygotes or compound heterozygotes for beta-thalassaemia, meaning that both beta-globin loci are affected^3^. Less commonly, only a single beta-globin locus is affected, the other being completely normal.^1^ The mild clinical characteristics of TI compared with TM major result primarily from three different mechanisms:^3^,^7^

- *Inheritance of a mild or silent beta-chain mutation*. Rather than a complete absence of beta-chain synthesis, the level of synthesis is subnormal. This leads to a smaller imbalance between the number of alpha-and beta-chains compared with an absence of beta-chains.
- *Co-inheritance of determinants associated with increased gamma-chain production*. The increased number of gamma-chains helps to neutralize the large proportion of unbound alpha-chains.
- *Co-inheritance of alpha-thalassaemia*. This helps to suppress the synthesis of alpha-chains, causing less of an alpha/beta-chain imbalance.

The phenotype of TI may result from the increased production of alpha-globin chains by a triplicated or quadruplicated alpha genotype associated with beta-heterozygosity.^8^–^10^ Table 1 shows β-globin mutations in TI and TM that have a direct effect on modifying the amount of excess alpha-chains, such as inheritance of abnormal alpha- or gamma-chain genes. Tertiary modifiers are polymorphisms occurring at loci involved in bone, iron and bilirubin metabolism which can affect clinical expression, although these are thought to be of relatively little importance. Recent studies of the JAK2 cytoplasmic tyrosine kinase, which has a vital role in signal transduction from several haemopoietic growth factor receptors, revealed a *V617F* mutation that was implicated in a variety of diseases mainly related to myelo-proliferative disorders including polycythaemia vera, essential thrombocythaemia, and idiopathic myelofibrosis. TI patients, however, do not show increased expression of this mutation.^11^

Environmental factors including social conditions, nutrition and the availability of medical care have also been implicated as key players in the phenotypic variability of TI.^12^

A number of studies have attempted to classify patients with TI according to the severity of their condition, although these studies have had only limited success.^13^,^14^ A recent study described the development of a phenotype scoring system that successfully sub-classified TI patients into three separate groups: mild, moderate or severe 15. The severity of TI was graded according to a number of clinical features, such as age at presentation, severity of anaemia, extent of growth retardation and bone marrow hyperplasia, blood transfusion requirements and need for splenectomy. This classification could prove useful for relating genotype to phenotype and for developing separate treatment guidelines for different disease severities. However, further studies would be required to confirm the reliability and utility of this approach.

Although the clinical phenotypes of thalassaemia minor, intermedia and major differ, there are some similarities. There is an increasing awareness of the need for accurate diagnosis in order to achieve optimal patient management and to avoid over or under treatment^3^,^16^. The accurate identification of TI versus thalassaemia minor and TM can be difficult if based on clinical presentation alone, although certain differentiating parameters have been established. In general, TI is characterized by Hb levels maintained around 7–10 g/dL without the need for regular blood transfusions, by more severe red blood cell (RBC) abnormalities than thalassaemia minor, by a varying degree of spleen enlargement, and by skeletal changes such as expansion of the facial bones and obliteration of the maxillary sinuses, which causes protrusion of the upper jaw.^5^,^6^,^16^ Criteria for differentiating TM from TI at presentation are summarised in Table 2.

## Pathophysiology

**Figure 1.**
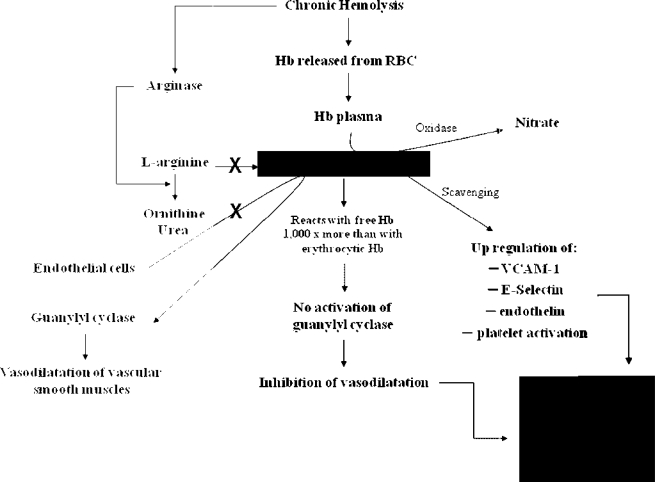
Nitric oxide dependent pathway leading to pulmonary hypertension and, possibly, thrombotic phenomena in thalassaemia intermedia patients (Hb = haemoglobin, RBC = red blood cell, PHT = pulmonary hypertension, VCAM = vascular cell adhesion molecule).

Three main factors are responsible for the clinical manifestations of TI: ineffective erythropoiesis, chronic haemolytic anaemia and iron overload. The severity of clinical sequelae primarily depends on the underlying molecular defects. Alpha-chains are highly unstable and precipitate within erythroid precursors in the bone marrow, causing membrane damage and cell death – this is ineffective erythropoiesis.^17^ Hypertrophy of erythroid marrow in medullary and extramedullary sites, a consequence of severe ineffective erythropoiesis, results in characteristic deformities of the skull and face and may also cause cortical thinning and pathological fractures of long bones.^16^,^18^ The degree of ineffective erythropoiesis is the primary determinant of the development of anaemia, while peripheral haemolysis of mature RBCs and an overall reduction in Hb synthesis are secondary. However, haemolysis per se has been linked to the development of a hypercoagulable state and pulmonary hypertension (PHT) in this patient population.^19^,^20^ Damaged circulating RBCs expose negatively charged phosphatidyl-serine residues through the ‘Flip-Flop’ phenomenon.^21^ These along with activation of platelets, endothelial cells and monocytes along with dysfunction of the coagulation system have been associated with thromboembolic phenomenon, especially in splenectomised TI patients^21^. Moreover, haemolysis carries a role in the dysregulation of nitric oxide (NO) homeostasis which is correlated with PHT and probably thrombotic phenomena (Figure 1).^22^

**Figure 2.**
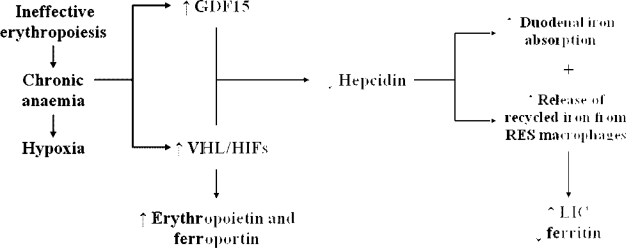
Pathophysiology of iron overload in patients with thalassaemia intermedia. GDF15 = growth differentiation factor 15; HIF = hypoxia-inducible transcription factor; RES = reticuloendothelial system; VHL = von Hippel-Lindau; LIC = liver iron concentration.

In contrast to patients with TM, in whom iron loading occurs mainly as a result of transfusion therapy, patients with TI accumulate iron primarily due to increased intestinal iron absorption.^23^ The chronic anaemia and ineffective erythropoiesis that are characteristic of TI are associated with reduced expression of hepcidin, a hepatic peptide that plays a central role in iron homeostasis.^23^ Recent studies indicate that growth and differentiation factor 15 (GDF15), secreted by erythroid precursors, is significantly increased in patients with TM, inhibiting hepcidin production.^24^ In addition, other studies indicate that hypoxia-inducible transcription factors (HIFs) control iron homeostasis by coordinating the downregulation of hepcidin and the upregulation of erythropoietin and ferroportin. Under normal conditions, HIFs play a useful role by mobilizing iron and supporting erythrocyte production in response to anaemia/hypoxia.^25^–^27^ However, the same mechanism may contribute to the harmful accumulation of iron in response to the chronic anaemia of TI. The combination of ineffective erythropoiesis (leading to increased GDF15) and chronic anaemia/hypoxia (altering the expression of HIF) results in hepcidin suppression, increased iron absorption and increased release of recycled iron from the reticuloendothelial (RE) system. This results in depletion of macrophage iron, and relatively low levels of serum ferritin. The situation in TI is similar to that seen in patients with hereditary hemochromatosis syndromes, which is characterized by impaired hepcidin production (Figure 2).

By contrast, in transfused TM patients iron is preferentially distributed to the RE system, stimulating ferritin synthesis and its release to the circulation and leading to high serum ferritin levels^28^. Blood transfusions modify hepcidin production by altering all three factors responsible for the clinical manifestations of TI described above namely: (i) by correcting anaemia, transfusion therapy will suppress ineffective erythropoiesis and the associated increase in GDF15; (ii) by correcting anaemia, blood transfusions will prevent the hypoxia responsible for increased HIF production and hypoxia-associated suppression of hepcidin; and (iii) increased body iron and transferrin saturation will stimulate hepcidin production. The result will be a relative increase in hepcidin production in polytransfused TM patients countering the hepcidin-inhibitory effects of anaemia and ineffective erythropoiesis. These considerations may explain the differences in iron homeostasis characterizing TM compared with untransfused TI patients. Thus, although the rate of iron loading may be slower than that in patients with TM, TI patients will inevitably suffer from iron overload which in turn can cause a number of serious complications including cardiac failure and endocrine abnormalities such as diabetes mellitus and hypogonadism.

## Clinical Sequelae

As a consequence of the aforementioned pathophysiology, several complications have been identified in patients with TI. Some of these complications have been termed unique to TI as compared to TM, especially in the splenectomised, transfusion naïve patient (Table 3). *Cholelithiasis* Gallstones are much more common in TI than in TM because of ineffective erythropoiesis and peripheral haemolysis. Recently, unrelated genetic factors such as uridine 5′-diphospho-alpha-D-glucose (UDPG) deficiency (Gilbert’s syndrome) have been reported to increase gallstone formation in patients with thalassaemia^29^. For this reason, the gallbladder should be inspected during splenectomy and a cholecystectomy performed if necessary, particularly if the patient is experiencing symptomatic gallstones. This should be undertaken to prevent cholecystitis, which can have serious consequences in the splenectomised patient.

### Extramedullary haematopoiesis

Extra-medullary haematopoiesis (EMH) is a compensatory mechanism where bone marrow activity increases in an attempt to overcome the chronic anaemia of TI, leading to the formation of erythropoietic tissue masses that primarily affect the spleen, liver and lymph nodes. These masses can be detected by magnetic resonance imaging (MRI). They may cause neurological problems such as spinal cord compression and paraplegia, and intrathoracic masses.^30^,^31^ Extramedullary haemato-poiesis can be managed by radiotherapy (since haematopoietic tissue is highly radiosensitive)^32^, transfusion therapy or hydroxyurea.^30^,^33^,^34^

### Leg ulcers

Leg ulcers are more common in older than in younger patients with TI. It is unclear why ulcers develop in some **patients** who are maintained at relatively low Hb levels and have the same amount of foetal Hb (HbF) as others in whom ulcers do not develop. The skin at the extremities of elderly TI patients can be thin due to reduced tissue oxygenation, and this makes the subcutaneous tissue fragile and increases the risk of lesions from minimal trauma. Once an ulcer has started to develop it is very painful and difficult to cure, although regular blood transfusions may provide some relief in persistent cases. Simple measures may be beneficial, such as keeping the patient’s legs and feet raised above the level of the heart for 1–2 hours during the day or sleeping with the end of the bed raised. Zinc supplementation^35^ and pentoxifylline, which alters the rheological properties of the RBCs,^36^ can help accelerate the healing of ulcers. Hydroxyurea also has some benefit, either alone or in combination with erythropoietin.^37^ In addition, the use of an oxygen chamber can provide moderate relief since tissue hypoxia may be an underlying cause of the ulceration.^38^

### Thromboembolic events

Taher et al.^39^ analyzed data from 8860 thalassaemia patients (6670 TM and 2190 TI). In this study thromboembolic events (TEE) occurred 4.38 times more frequently in TI than TM patients, with more venous events occurring in TI and more arterial events occurring in TM. In another series of TI patients, 24 patients (29%) developed either deep vein thrombosis (DVT), pulmonary embolism, or portal vein thrombosis during a 10-year follow up.^40^ In a recent survey involving nine Italian pediatric thalassaemia centres, TEE was observed in 4% of 683 patients with TM and in 9.6% of 52 patients with TI.^41^ Studies collectively revealed that the incidence of TEE is higher in splenectomised patients.^39^,^40^ The incidence of overt stroke in TI patients is low;^8^ however, a study done to assess the rate of asymptomatic brain damage in patients with benign haemoglobinopathies reported that 37.5% of patients with TI showed silent brain ischemia.^42^

### Pulmonary hypertension and congestive heart failure

Pulmonary hypertension (PHT) is prevalent in patients with TI (59.1%),^20^ and is thought to be the primary cause of congestive heart failure in this patient population. A retrospective analysis showed that splenectomised females with significant anaemia, thrombocytosis and elevated ferritin levels, were at greatest risk for developing PHT.^43^ Preliminary results have demonstrated that PHT is reversible by blood transfusion and treatment with aspirin and warfarin. Several echocardiographic studies have confirmed that cardiac ejection fraction is rarely affected in TI.^44^ Nevertheless, patients with TI often have an increased cardiac output, and left ventricular wall dimensions proportional to the dilutional volume overload secondary to chronic anaemia.^45^

As anaemia and iron overload are uncommon in well-transfused and chelated TM patients, they are likely to be at the heart of the pathophysiology of PHT. A recent study demonstrated a significant correlation between iron overloading in the liver and pulmonary artery systolic pressure independent of left ventricular filling pressures.^46^ Regular transfusion and iron chelation therapy is therefore indicated in TI patients who are well-stratified according to the early detection of PHT indices. Sildenafil has also been successfully used to treat PHT,^47^ although data from large patient numbers are lacking in TI.

### Pregnancy and related complications

Women with TI may have spontaneous successful pregnancies although complications during pregnancy may occur^48^. The chronic anaemia of TI can cause an increase in spontaneous abortions, pre-term labour and intrauterine growth retardation, while endocrine complications due to haemo-siderosis are common^49^. The course and outcome of 19 pregnancies was assessed in 16 women with thalassaemia, including four with TI^50^. All pregnancies were uneventful and elective Caesarean section was performed in each case. The mean birth weight of the babies was 3000 g and all were normal except for one case of omphalocele. The largest study to date assessed 44 TI women who had 83 pregnancies, all spontaneous, 30 from Lebanon and 53 from Italy.^51^ These pregnancies resulted in 20.5% abortions, 77.1% live-births and 2 intrauterine foetal deaths at 26 and 36 weeks’ gestation. The mean gestational age at delivery was 36.5 weeks and mean birth weight was 2551 g. In pregnancies progressing > 20 weeks’ gestation, pre-term delivery and intrauterine growth restriction (IUGR) were noted in 31.8% and 24.2% of cases, respectively. In those complicated by IUGR, Caesarean delivery (CS) rate was 87.5%. Two women (Italy) developed severe alloimmune haemolytic anaemia. One progressed to cardiac failure at 35 weeks’ gestation and had CS. The other underwent CS for IUGR and non-reassuring foetal heart monitoring and was scheduled for a splenectomy postpartum. Worsening alloimmune anaemia also developed in 2 women from Lebanon who required splenectomy within eight weeks postpartum. Transfusion was required in 35/44 women during pregnancy (79.5%), with 27.3% requiring transfusion during pregnancy for the first time. The lowest mean Hb level was 6.7±2.0 *vs.* 8.3±1.2 g/dL in Lebanon and Italy respectively. The average ferritin level before pregnancy was 885.2 ± 658.9 *vs.*1232.8 ± 902.9 μg/L after pregnancy. In total, CS was performed in 48 pregnancies (72.7%), the indications being elective (41.7%), repeat (31.2%) and obstetrical (27.1%). Pregnancy outcome was similar between Lebanon and Italy with the exception of a significantly higher rate of live births in Italy.^51^

Folic acid deficiency is common in TI and occurs due to poor absorption, low dietary intake, or, most significantly, an increased demand for folic acid from active bone marrow. This is a particular concern in pregnancy since deficiency can cause neural tube defects, such as spina bifida, in the growing foetus. During pregnancy, women with TI should therefore be given oral folic acid supplementation (around 1 mg/day), and should be carefully monitored in order to assess the need for transfusion therapy and to avoid haemodynamic compromises. The major fear of initiating transfusions during pregnancy is the development of alloantibodies. These can aggravate anaemia and progress into severe haemolytic anaemia refractory to transfusions and thus increase the complication rate. CS may be required in TI patients due to the associated cephalopelvic disproportion secondary to skeletal deformity and short stature, especially in non-transfused women. Splenectomy is usually performed in TI women for decreased levels of haemoglobin, hyperactivity of the spleen, leukopenia and symptomatic thrombocytopenia.^5^,^6^

## Management

There are a number of options currently available for managing patients with TI, including splenectomy, transfusion therapy, iron chelation therapy, modulation of HbF production and haematopoietic stem cell transplantation.

### Splenectomy

As per expert opinion, the current indications for splenectomy in TI include growth retardation or poor health, leukopenia, thrombocytopenia, increased transfusion demand, or symptomatic splenomegaly.^6^ Clinical observations, however, have suggested that splenectomy in TI can contribute to an increased susceptibility to TEE and PHT^39^,^40^,^43^. This calls for a review of splenectomy as a procedure of choice, especially with its potential role in increasing TI-related complications and the inherent risk of infection associated with the procedure even for individuals without haematological disorders.^52^

### Transfusion and iron chelation therapy

In patients with TM, a remarkable improvement in life expectancy and prevention of morbidity has been achieved in recent decades 53. This is mainly attributed to improved methods of blood transfusion, better understanding of iron toxicity, and evolution in iron chelation therapy.^53^ On the other hand, TI has been regarded as a clinical entity with limited complicationsso as the general approach was to avoid early blood transfusions and the associated need for chelation therapy. However, increasing evidence is delineating the benefit of transfusion therapy in decreasing the incidence of complications as PHT, TEE, and EMH, to name a few.^30^,^39^,^54^ Thus although the common practice was to initiate transfusion when complications ensue, it may be worthwhile start transfusion therapy earlier as a preventive approach which will also help alleviate the increased risk of alloimmunisation with delayed initiation of transfusion.^55^ Although earlier introduction of blood transfusions will increase the rate of iron accumulation, effective methods of iron chelation are now available 56,57, and the benefits of transfusion therapy would greatly outweigh the cost and inconvenience of iron chelation therapy.

The initiation of iron chelation therapy in patients with TI depends not only on the amount of excess iron, but also on the rate of iron accumulation, the duration of exposure to excess iron and various other factors in individual patients. A direct assessment of LIC is recommended, either by biopsy or by a non-invasive method such as R2 MRI. Where LIC measurement is not possible, threshold serum ferritin values of 400–500 ng/mL (which are lower than those generally accepted in patients with TM) could be considered as an indicator for initiation of iron chelation therapy. Chelation therapy should generally be initiated if LIC exceeds 7 mg/g dry weight of liver tissue, however lower levels of LIC for initiation of chelation therapy must be considered particularly now with the availability of oral iron chelators.^6^

### Modulation of foetal haemoglobin production

Increasing the synthesis of HbF can help alleviate anaemia and therefore improve the clinical status of patients with TI 58. Production of HbF is reactivated during recovery from marrow suppression after treatment with cytotoxic drugs, therefore it is postulated that these agents may alter the pattern of erythropoiesis and increase the expression of gamma-chain genes. Several cytotoxic agents with this effect have been identified, including cytosine arabinoside and hydroxyurea.^59^–^61^ Recently published results from Iran, evaluating six years of hydroxyurea therapy in transfusion dependent patients with TI, are encouraging. A significant decrease in the need for blood transfusions was observed in many patients; the need was completely obviated in some patients.^62^ Erythropoietin has also been shown to increase HbF levels in some patients with TI^58^. Preliminary trials with intravenous and oral butyric acid derivatives have shown increases in foetal and total Hb levels in patients with TI,^63^–^66^ and the acceptable safety profile of these agents makes them promising therapeutic targets. It is unclear how butyrates stimulate gamma-globin production or why some patients respond to treatment while others do not.

However, the overall trial results with HbF-stimulating agents are somewhat disappointing. Studies using combined treatments have shown greater promise than the individual agents alone^67^. Further clinical evaluation is required to clarify the value of this approach, especially in view of the reduced oxygen delivery capacity of HbF, as this might favour the implementation of a target Hb level higher than 10 g/dL in response to increased need (e.g. PHT, coronary heart disease and chronic obstructive pulmonary disease (COPD)) and an increased ratio of HbF/HbA.

### Haematopoietic stem cell transplantation

Haematopoietic stem cell transplantation (HSCT), where the marrow of an affected patient is replaced from the stem cells of an uaffected donor, is an established treatment for beta-thalassaemia. Although successful HSCT can offer a cure, it can be unsuccessful (e.g. if the thalassaemia returns), may lead to complications (e.g. graft-versus-host disease, growth impairment, neurological complications), and can even result in death;^68^–^71^ the risk for a failed transplantation depends primarily on the health and age of the patient. The decision as to which patients are eligible for transplantation is complex and is related to both the quality of life and expected survival-time of the transplanted patient, when compared with supportive care only. This is particularly relevant in patients with TI, especially in those who are only mildly affected. Due to the risks involved, transplantation is considered appropriate only for patients with a human leukocyte antigen (HLA)-matched donor, which comprises only 30–40% of all beta-thalassaemia patients, at most.^72^ As HLA type is genetically determined, there is a 25% chance that any two siblings will be a match.

## Conclusion

Our understanding of the molecular and pathophysiological mechanisms underlying the disease process in patients with TI has substantially increased over the past decade. Today, a large body of evidence documents the incidence and consequences of the different clinical complications associated with the disease. Although there are still currently no clear guidelines for the optimal management plan of this disease entity, the emerging body of evidence looks promising.

## Declaration Of Interest

The authors report no conflicts of interest. The authors alone are responsible for the content and writing of the paper.

This study did not receive external funding.

## Figures and Tables

**Table 1.** Prevalent beta-globin mutations in thalassaemia intermedia and thalassaemia major in patients of Mediterranean origin.^7^

**Mutation**	**Thalassemia Intermedia n(%)**	**Thalassemia major n(%)**
cd 39 C->T	72 (24)	136 (53.5)
IVSI-110 G->T	52 (17)	58 (23)
IVSI-6 T->C	94 (31.5)	16 (6.3)
IVSI-1 G->A	8 (2.7)	19 (7.4)
IVSII-1 G->A	14 (4.7)	10 (3.9)
IVSII-745 C->G	11 (3.7)	9 (3.5)
−101 C->T	10(3.3)	-
cd6 - A	10(3.3)	3 (1.2)
−87 C->G	8 (2.7)	-
δβSiciliana	13 (4.3)	-
Lepore Boston	2 (0.7)	-
IVSI-5 G->A	-	1 (0.4)
IVSI-5 G->C	1 (0.3)	-
IVSII-844 G->C	1 (0.3)	-
IVSI-2 T->A	1 (0.3)	-
cd 44 - C	-	1 (0.4)
cd 8 - AA	1 (0.3)	1 (0.4)

Total	298 (100)	254 (100)

**Table 2.** Criteria to differentiate thalassaemia major from intermedia at presentation.

**Parameter**	**Thalassaemia major more likely**	**Thalassaemia intermedia more likely**
**Clinical**		
Presentation (years)	<2	>2
Liver/spleen enlargement	Severe	Moderate to severe
**Haematological**		
Hb level (g/dL)	6–7	7–10
HbF (%)	>50	10–50 (may be up to 100%)
HbA2 (%)	<3.5	>3.5
**Genetic**		
Parents	Both carriers of high HbA2 β-thalassaemia	One or both atypical carriers: – High HbF β-thalassaemia – Borderline HbA2
**Molecular**		
Type of mutation	Severe	Mild/silent
Co-inheritance of α-thalassaemia	No	Yes
Hereditary persistence of HbF	No	Yes
δβ-thalassaemia	No	Yes
Gγ Xmn1 polymorphism	No	Yes

Hb = haemoglobin; HbF = foetal haemoglobin

**Table 3.** Prevalence of complications in thalassaemia intermedia vs. major.^5^

**Complication (% of patients affected)**	**Thalassaemia intermedia**		**Thalassaemia major**	

	*Lebanon*	*Italy*	*Lebanon*	*Italy*
	(n = 37)	(n = 63)	(n = 40)	(n = 60)
Splenectomy	90	67	95	83
Cholecystectomy	85	68	15	7
Gallstones	55	63	10	23
Extramedullary haemopoiesis	20	24	0	0
Leg ulcers	20	33	0	0
Thrombotic events	28	22	0	0
Cardiopathy*	3	5	10	25
Pulmonary hypertension^†^	50	17	10	11
Abnormal liver enzymes	20	22	55	68
HCV infection	7	33	7	98
Hypogonadism	5	3	80	93
Diabetes mellitus	3	2	12.5	10
Hypothyroidism	3	2	15	11

* Fractional shortening < 35%.

† Defined as pulmonary artery systolic pressure > 30 mmHg; a well-enveloped tricuspid regurgitant jet velocity could be detected in only 20 patients, so frequency was assessed in these patients only.
